# *Streptococcus pneumoniae* Type 1 Pilus – A Multifunctional Tool for Optimized Host Interaction

**DOI:** 10.3389/fmicb.2021.615924

**Published:** 2021-02-09

**Authors:** Stefan Ness, Markus Hilleringmann

**Affiliations:** FG Protein Biochemistry & Cellular Microbiology, Department of Applied Sciences and Mechatronics, Munich University of Applied Sciences, Munich, Germany

**Keywords:** pilus, bacteria, *Streptococcus pneumoniae*, architecture, sortase, virulence, host-interaction, mechanobiology

## Abstract

*Streptococcus pneumoniae* represents a major Gram-positive human pathogen causing bacterial pneumonia, otitis media, meningitis, and other invasive diseases. Several pneumococcal isolates show increasing resistance rates against antibacterial agents. A variety of virulence factors promote pneumococcal pathogenicity with varying importance in different stages of host infection. Virulence related hair-like structures (“pili”) are complex, surface located protein arrays supporting proper host interaction. In the last two decades different types of pneumococcal pili have been identified: pilus-1 (P1) and pilus-2 (P2) are formed by the catalytic activity of sortases that covalently assemble secreted polypeptide pilin subunits in a defined order and finally anchor the resulting pilus in the peptidoglycan. Within the long pilus fiber the presence of intramolecular isopeptide bonds confer high stability to the sequentially arranged individual pilins. This mini review will focus on *S. pneumoniae* TIGR4 P1 molecular architecture, the subunits it builds and provides insights into P1 sortase-mediated assembly. The complex P1 architecture (anchor-/backbone-/tip-subunits) allows the specific interaction with various target structures facilitating different steps of colonization, invasion and spreading within the host. Optimized pilin subunit confirmation supports P1 function under physiological conditions. Finally, aspects of P1- host interplay are summarized, including recent insights into P1 mechanobiology, which have important implications for P1 mediated pathogenesis.

## Introduction: *Streptococcus pneumoniae* a Major Human Pathogen Expressing Different Types of Pili

*Streptococcus pneumoniae* (the pneumococcus) is a human commensal bacterium that can cause lethal diseases like pneumonia, septicemia, and meningitis. As major human pathogen, it provokes high morbidity and mortality rates especially in children and the elderly. Licensed pneumococcal vaccines are not covering all relevant virulent strains and an increasing number of antibiotic resistant isolates makes treatment challenging (Subramanian et al., 2019). Novel, broad-spectrum vaccination strategies and new antibacterials are of utmost importance to combat *S. pneumoniae*. The switch from a human commensal to an invasive pneumococcal pathogen and its disease causing capacity in various host niches is an area of intense study (Loughran et al., 2019). A multiplicity of differentially regulated cell surface located molecules mediate the complex interplay of *S. pneumoniae* and the human host. This includes several types of high molecular weight protein assemblies, so-called pili, which promote pneumococcal virulence. Although *S. pneumoniae* was first isolated by Pasteur in 1881, pneumococcal pili were discovered only at the beginning of the 21st century. Besides a recently described type IV competence pilus (Laurenceau et al., 2013; Muschiol et al., 2019), pneumococcal isolates express two variants of Gram-positive, sortase cross-linked multi-subunit pili [“pilus-1 (P1) and pilus-2 (P2)”] (Barocchi et al., 2006; Bagnoli et al., 2008). In this review, we focus on the assembly and particular architecture of pneumococcal P1 and derived from that summarize its role as multifunctional host-interaction tool. This indicates a virulence-mediating role of P1 at different phases of pneumococcal diseases and an optimized P1-structure for various host environments.

## Subunits and Sortase-Mediated Assembly of Pneumococcal Pilus-1

**FIGURE 1:**
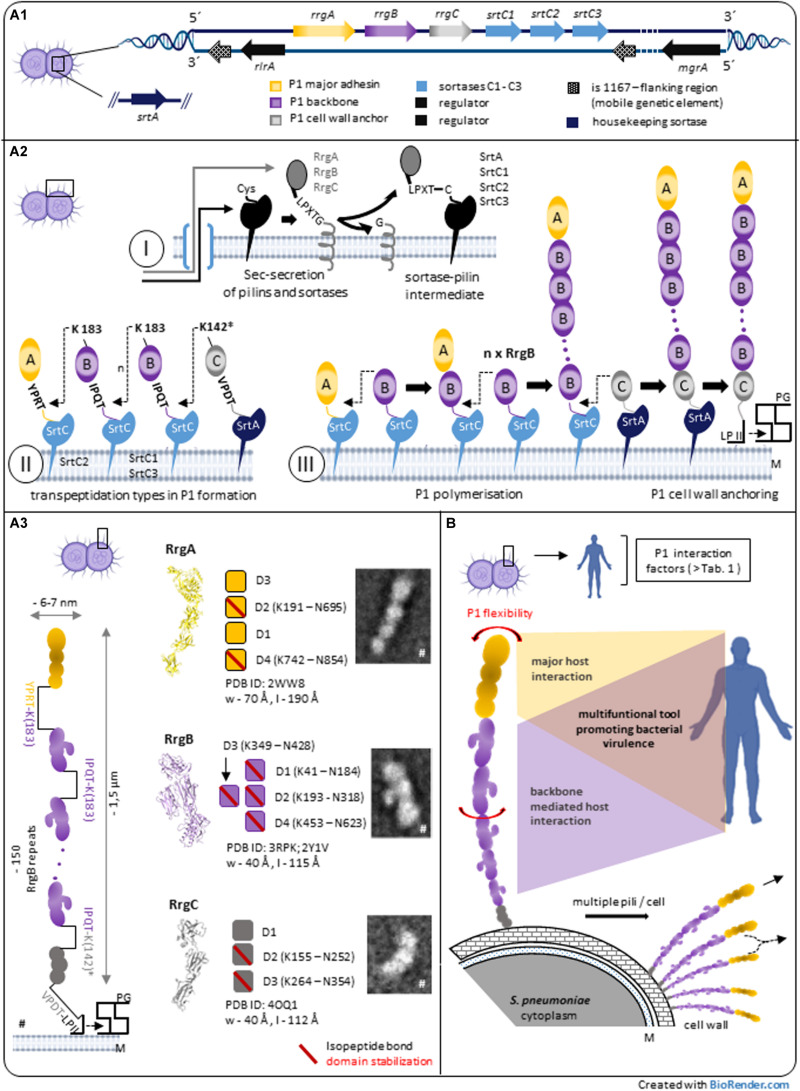
Pilus-1 of *Streptococcus pneumoniae* – assembly, architecture and structure-derived properties. **(A1)** *Streptococcus pneumoniae* TIGR4 pilus-island 1 (PI-1) and related genes involved in P1 biosynthesis. **(A2)** Model of sortase-mediated assembly of pneumococcal pilus-1. *A2-I:* P1-pilins and relevant sortases are expressed in the cytoplasm, exported by the Sec secretion system and surface located via a C-terminal hydrophobic stretch in the bacterial plasma membrane. The initial step of sortase-catalyzed transpeptidation is a pilin CWSS specific hydrolysis between Thr and Gly of particular variants of the LPXTG motif via the enzyme’s active site Cys resulting in a sortase-pilin thioacyl intermediate. *A2-II:* C-terminal pilin Thr in the activated sortase-pilin complex (A2-I) is linked to a conserved Lys in the next P1 subunit of the growing pilus. P1 heterotrimeric formation is a complex process involving the sequential incorporation of RrgA, RrgB, and RrgC via pilin specific PI-1 class C-sortases. Shown are details of individual P1 sortase-pilin intermediates and respective Lys residues involved in transpeptidation reactions during P1 formation. Simulation results combined with existing biochemical and genetic data of several groups suggest the following PI-1 class C-Srt specificities (Naziga and Wereszczynski, 2017): SrtC1 is the primary Srt for RgB polymerization; in the absence of RrgB, SrtC1 might add RrgA, and RrgC to the pilus. SrtC3 shows a pronounced RrgB crosslinking activity when compared to RrgA and RrgC although less distinctive than SrtC1-RrgB. The first substrate of SrtC2 is RrgA linking the P1 adhesin to the RrgB backbone. Apart from background activity of SrtC-1, housekeeping SrtA is defined as principal RrgC-related sortase. *A2-III:* P1 assembly is a biphasic process of P1 polymerization and final, lipid II-mediated, cell wall anchoring. **(A3)** P1 heterotrimeric architecture and pilin characteristics. P1 is a thin, long filament composed of a proximal RrgC anchor, n-serial RrgB backbone molecules and a distal RrgA adhesin. TEM images and high-resolution structures of P1-pilins (RrgA, RrgB, and RrgC) identify elongated molecules containing intramolecular, isopeptide bond stabilized domains. A distinctive feature of RrgB is a lateral subunit (D3) that is involved in host interaction (^#^parts adapted from Hilleringmann et al., 2009). **(B)** P1 expressing pneumococci exhibit multiple pilus filaments on the bacterial surface functioning as single fiber or potential P1 aggregations. In addition to P1 subunit stabilization **(A3)**, the entire P1 fiber shows a pronounced bendability. Longitudinal rotation of serial arranged RrgB subunits along the P1 axis suggest additional optimization of P1 architecture. P1 host interaction is complex, mainly mediated by terminal adhesin RrgA. Force induced RrgB host factor interaction indicate a functional role of P1 backbone in addition to its role as stabilized stalk exposing terminal P1 adhesin. *Potential nucleophilic RrgC Lys residue. CWSS, cell wall sorting signal; LP II, lipid II; M, bacterial plasma membrane; PG, peptidoglycan; P1, Pilus-1; Srt, Sortase.

While typical Gram-negative pili are formed by non-covalent interactions between pilins, the covalent assembly of Gram-positive pili is catalyzed by specific sortases involving pilus subunit polymerization and cell wall anchoring of the resulting pilus fiber (Telford et al., 2006; Hendrickx et al., 2011; Hospenthal et al., 2017). *Streptococcus pneumoniae* TIGR4, belonging to the highly invasive pneumococcal serotype 4, is a patient isolate were P1 was initially identified (Barocchi et al., 2006) that represents an important reference strain studying P1 biology. Major components involved in pneumococcal TIGR4 P1 formation are clustered in a defined genetic region [pilus island 1 (PI-1)] that encodes 3 P1 specific class C sortases (SrtC-1, SrtC-2, SrtC-3), 3 P1 subunits (RrgA, RrgB, and RrgC) and a transcriptional regulator (RlrA) (Figure 1-A1). Analysis of strain collections indicates that only a subset of pneumococcal isolates expresses P1 (∼ 30%) (Dzaraly et al., 2020). Electron microscopic analysis allowed the initial visualization of pili on the surface of negative stained TIGR4 isolates. Negative staining procedure improves contrast for better visualization of few nm thin pilus filaments. The explicit identification of PI-1 encoded pilins within P1 required specific immuno-labeling strategies and resulted in heterotrimeric P1 working models with RrgB as major pilin and 2 minor pilins (RrgA and RrgC) (Barocchi et al., 2006; LeMieux et al., 2006, 2008; Hilleringmann et al., 2008; Fälker et al., 2008).

Similar to other Gram-positive pilus systems, the current model of P1 formation involves pilus subunit- and Srt-synthesis in the cytoplasm and respective surface localization followed by a biphasic process of pilus-polymerization and -anchoring to the bacterial cell wall (Khare and Narayana, 2017; Figure 1-A2): After their cytoplasmic expression, respective N-terminal signal sequences of P1-pilins and -sortases allow Sec-translocation into the exoplasmic zone. A C-terminal hydrophobic stretch functions as membrane anchor and promotes their embedding into the lipid bilayer. Since P1 discovery, a main research focus lies on the respective role of PI-1 sortases for P1 assembly, including molecular details of the catalyzed transpeptidation and Srt-regulation: during P1 polymerization, PI-1 individual class C sortases recognize particular variants of LPXTG motif pentapeptide-cell wall sorting signals (CWSSs) at the C-terminus of individual P1 pilins as described for other Gram-positive pilus systems. The sortase- catalytic activity hydrolyses between the CWSS threonine and glycine residues by an initial nucleophilic attack of the threonine’s carbonyl carbon atom via the enzyme’s active site cysteine residue (Figure 1-A2/I). The Thr C-terminus in the formed sortase-pilin thioacyl intermediate is linked to a conserved lysine ε-amino group within the pilin motif of the next P1 subunit of the growing pilus, RrgB K183 (within RrgB pilin motif WXXXVXVYPK) and finally specific lysine residue of RrgC (structural analysis suggest Lys K142 as respective nucleophilic residue of RrgC, although experimental proof is missing; (Figure 1-A2/II–III; LeMieux et al., 2006, 2008; Fälker et al., 2008; Manzano et al., 2008; Neiers et al., 2009a,b; El Mortaji et al., 2012b; Shaik et al., 2014). The data support a gradual selectivity of the three PI-1 SrtC isoforms to individual P1 pilin CWSSs during P1 polymerization (Figure 1-A2/II). Differences in CWSSs (RrgA: YPRTG; RrgB: IPQTG; RrgC: VPDTG) and resulting conformational changes might direct binding preferences of respective PI-1 SrtCs. Recent molecular dynamics simulations suggest a key role of the middle amino acid in the five-residue pilin CWSSs for selective and specific sorting signal targeting of the individual SrtCs (Naziga and Wereszczynski, 2017).

The second step in P1 biosynthesis is the covalent linkage of the polymerized P1 to the bacterial cell wall. Deletion of minor pilin RrgC leads to P1 polymers secreted into the supernatant and identifies RrgC as P1 cell wall anchor molecule (Hilleringmann et al., 2009; Shaik et al., 2014). The primary sortase for Lipid II-mediated RrgC anchoring to peptidoglycan is the pneumococcal housekeeping sortase SrtA (Shaik et al., 2014; Naziga and Wereszczynski, 2017). This is in agreement with SrtA pilus-anchoring activity in other Gram-positive pili systems.

Both, PI-1 class C-Srts and SrtA are central elements for P1 formation. Their coordinated activity seems essential for proper P1 assembly. Interestingly, all P1 class C Srts contain a N-terminal lid-region that covers the active catalytic site triad region constituted of His, Arg, and Cys that is missing in the respective housekeeping sortase and suggest a regulatory element of SrtCs activity. Whereas initial results indicate a flexible, “mobile” lid behavior of SrtC-1 in solution (Manzano et al., 2009), recent data propose a rather rigid SrtC-lid condition in the absence of substrate with a potential negative regulatory function (Jacobitz et al., 2016).

Some important questions regarding the mechanistics of Srt-mediated P1 fiber assembly are ambiguous: (i) Precise chronology of P1 formation including the factors determining the starting of the biosynthesis until final anchoring of P1 to the cell wall is missing. (ii) Sequence details of P1 pilin incorporation: although major backbone RrgB polymerization was observed in a RrgA deletion strain background (Nelson et al., 2007), the “tip first rule” proposing a first transpeptidation reaction between the terminal pilus adhesin and a pilin backbone molecule [as described for the well studied heterotrimeric SpaA pili of *Corynebacterium diphtheriae* (Ramirez et al., 2020)] seems reasonable for other Gram-positive pili systems, but needs detailed experimental proof in the P1 system. (iii) Regulation of P1 length: based on a model of “pilus chain terminator role” of anchor pilin SpaB in *C. diphtheriae* SpaA pilus (Mandlik et al., 2008), it should be investigated whether the step of RrgC anchor incorporation into the SrtC-activated RrgB_*n*_RrgA fiber has a similar role in defining P1 final length. Importantly proper regulation of P1 RrgB mediated length determines the relative position of major adhesin RrgA and might be crucial for P1 functionality as recently demonstrated in other Gram-positive pilus systems (Chang et al., 2019).

TIGR4 bacterial surface is covered with multiple copies of P1 (Barocchi et al., 2006; Hilleringmann et al., 2009). The regulation of the spatio-temporal P1 distribution, including signal sequence directed cellular export and surface-positioning of pilins and Srts (Spirig et al., 2011), plus the precise interplay between P1 assembly and peptidoglycan metabolism is not understood. Topological studies describe a specific pattern of P1 subunits on the bacterial surface (“symmetric P1 foci”) indicating a non-homogenous P1 distribution (Fälker et al., 2008).

In addition to specific control mechanisms focused on proper P1 biosynthesis, signals from general regulatory networks of the complex interplay between *S. pneumoniae* and the human host are supposed to influence P1 expression (Kreikemeyer et al., 2011; Gómez-Mejia et al., 2018). Transcriptional P1 regulation seems to be of major importance: besides a suggested negative regulation by RrgA (Basset et al., 2011), PI-1 was described to be positively controlled by a RlrA feedback loop (Basset et al., 2012). In addition to central *rlrA*, other PI-1 positive regulators were described (Hava et al., 2003; Kreikemeyer et al., 2011). Within the growing list of negative regulators (Basset et al., 2011; Kreikemeyer et al., 2011; Herbert et al., 2015), some of them might require additional validation (Basset et al., 2017). Detailed investigations with extending systems that reflect the *in vivo* situation as closely as possible will help to further clarify the overall context of pneumococcal P1 regulation during host colonization and invasion (Pancotto et al., 2013; Figueira et al., 2014). The described absence of P1 expression below 31°C indicates the importance of host environmental factors on P1 regulation (Basset et al., 2017). Analysis of PI-1 containing isolates identified sub-populations of P1-expressing and – non expressing bacteria. Angelis et al. (2011), Basset et al. (2011) which might be a compromise balancing P1-related advantages like improved human colonization and –potential disadvantages of P1 induced immunogenicity and biosynthesis costs (Binsker et al., 2020; Iovino et al., 2020).

## Design of Multimeric TIGR4 Pilus-1 Architecture Reflects an Optimized Interaction Tool

High resolution analysis of surface digested individual pili allowed a detailed analysis of heterotrimeric P1 architecture and pilin stoichiometry, overcoming interferences and reducing complexity of respective cell bound-P1 study objects. TIGR4 P1 basic structure is a long, only ∼ 6–7 nm-wide filament composed of a multiple repetition of RrgB backbone molecules, with one RrgA adhesin and one RrgC minor pilin at the P1 distal and proximal end, respectively, associated in a head-to-tail covalently linked fashion. Scanning TEM analysis indicated that a 1.5-μm-long pilus comprises approximately 150 RrgB monomers in which a nose-like protrusion in each RrgB subunit defines the polarity of the fiber (Hilleringmann et al., 2009; Figure 1-A3). Adjacent P1 filaments on the cell wall surface can tangle or form bundles. Aggregations of different length pili with terminal RrgA, could explain the described P1 coiled-coiled phenotype with surface located RrgA molecules (Hilleringmann et al., 2008). RrgA aggregates were described as better TLR2 agonist *in vitro* when compared to RrgA monomer (Basset et al., 2013). Respectively, clustered P1 RrgA on the bacterial surface might be better recognized by the innate immune receptor TLR2 *in vivo*.

High resolution crystal structures of all P1-pilins have been resolved (Figure 1-A3): major adhesion RrgA (PDB: 2WW8) represents a four domain, elongated molecule, carrying segments of eukaryotic origin important for host interaction, mainly mediated by RrgA D3 domain (Izoré et al., 2010; Moschioni et al., 2010). The backbone RrgB (PDB: 3RPK and 2Y1V) subunit displays a four domain fold (Paterson and Baker, 2011; El Mortaji et al., 2012a). Interestingly a domain (D3) is arranged laterally of the RrgB middle domain (D2) which constitutes a special structural feature among Gram-positive major pilins (Young et al., 2014) and presumes a specific P1 adaptation for optimized interaction (Becke et al., 2019). Reconstruction of P1 architecture by fitting of the P1 fiber with high resolution crystal structure of RrgB D2-D4 molecules and RrgB D1 computer model data showed a rotation along the longitudinal P1 axis between two neighboring RrgB subunits of about 17–22° (Spraggon et al., 2010). A resulting different spatial domain arrangement of sequential RrgB molecules might further improve host interaction via multiple linearly arranged RrgB molecules (Figure 1-B), like a RrgB D3 mediated collagen interaction (Becke et al., 2019). Anchor subunit RrgC is the smallest P1 pilin and folds into three independent domains (PDB: 4OQ1) (Shaik et al., 2014). P1 pilins show a characteristic domain-based architecture that incorporated into P1 filament resemble a “beads on a string” macroscopic phenotype. Like described for other Gram-positive pilus systems, the existence of eukaryotic IgG-like subdomain variants within all P1 pilins (Izoré et al., 2010; Paterson and Baker, 2011; Shaik et al., 2014) suggest an adaptation strategy for proper P1 mediated host factor interaction while minimizing adverse host immune system responses by mimicry of eukaryotic immune system elements (Krishnan et al., 2007; Shaik et al., 2014).

Structural analysis of Gram-positive pilins revealed domain stabilization via intramolecular isopeptide bond formations (Kang et al., 2007; Baker et al., 2015). P1 subunits show different numbers of stabilized individual domains [RrgA: 2 (Izoré et al., 2010); RrgB: 4 (El Mortaji et al., 2010, 2012a; Paterson and Baker, 2011), and RrgC: 2 (Shaik et al., 2014); Figure 1-A3] that might reflect individual pilin adaptation to generate an optimized P1 multimeric tool able to withstand mechanical perturbations induced during host interaction and supporting P1 assembly. Recent single-molecule force spectroscopy data propose structural concepts to protect covalent bonds of Gram-positive pilins from cleavage under mechanical challenge (Echelman et al., 2016).

In addition to pure pilus mechanical stability and similar to other Gram-positive pili, functional P1 mediated host interaction requires a certain extent of flexibility of the P1-filament to efficiently target host structures (Figure 1-B). Macroscopic analysis of P1 phenotype of cell-bound and isolated pili revealed a substantial degree of fiber bendability (Barocchi et al., 2006; Hilleringmann et al., 2009). The respective percental contribution of the flexible linker regions between individual P1 pilins within the fiber and subunit derived flexibility, i.e., interdomain malleability, needs further investigation and might be P1 subunit specific (Spraggon et al., 2010; El Mortaji et al., 2012a).

## Multifunctional Pneumococcal Type-1 Pili Promote Various Interaction Scenarios

The deletion of P1 attenuated the virulence of respective clinical isolates in mouse models of pneumococcal infection (Barocchi et al., 2006). Work by several groups indicate a significant involvement of major adhesin RrgA in various steps of pneumococcal colonization and virulence addressing different host niches, which is described in a recent, comprehensive review (Iovino et al., 2020) and summarized in Table 1A). RrgA mediates in a RrgB backbone independent way, adherence to host epithelial cells, potentially promoting initial host colonization (Nelson et al., 2007). Dose-dependent interactions of RrgA with ECM molecules (fibronectin, laminin, and collagen I), not seen for RrgB and RrgC, indicate specific binding to host cell associated molecules (Hilleringmann et al., 2008). In addition to static ELISA approaches, data from single molecule force spectroscopy specify a two-domain binding mechanism for RrgA with Fn, that suggest a P1-mediated bacterial adaptation to keep contact with host tissue surface in shear force environments (Becke et al., 2018). RrgA was found to interact with complement receptor 3 (CR3) that promotes CR3 –mediated uptake of *S. pneumoniae* expressing P1-RrgA by macrophages and results in pneumococcal stimulation of macrophage motility. Additionally, expression of P1 RrgA together with host expression of CR3 affects virulence and spreading of the pathogen from local sites to the bloodstream in mice (Orrskog et al., 2012). Data of a RrgA D3 dependent TLR2-activation and induction of inflammatory TNF-α responses (Basset et al., 2013) suggest further P1-mediated interaction with components of the innate immune system. Overall implications of the RrgA-mediated TLR2-activation of the host immune response, a potential limiting consequence of P1 expression on pneumococcal fitness and virulence, need further investigation. The described more potent activation of TLR2 by oligmeric forms of RrgA might be mediated by aggregated monomeric RrgA of individual P1 fibers on the pneumococcal surface (Figure 1-B). Lethal meningitis caused by *S. pneumoniae* requires the penetration of the blood-brain-barrier (BBB) by the bacteria (Iovino et al., 2016b): an interaction of RrgA with polymeric immunoglobulin receptor (pIgR) and platelet endothelial cell adhesion molecule 1 (PECAM-1), two BBB endothelial receptors, was found to promote the entry of bacteria into the brain and meningitis development (Iovino et al., 2017). Interestingly, a subpopulation of spherical single pneumococci, that do not express DivIVA, with surface-located P1-RrgA seem to be favored to cross the BBB. In addition to the expression of RrgA, the small size of these pneumococcal variants might explain easier penetration of the BBB (Iovino et al., 2016a). In addition, recent data imply that meningitis related neuronal death is mediated by an interaction of RrgA and pneumolysin with β-actin of human neurons (Tabusi et al., 2020). The formation of biofilms promotes the persistence of pathogenic bacteria on patient’s tissues and overall virulence (Muhammad et al., 2020). *In vitro* screening for biofilm-altered TIGR4 mutants identified RrgA as potential molecule favoring bacterial interaction (Muñoz-Elías et al., 2008).

**TABLE 1:** *Streptococcus pneumoniae* pilus-1 mediated interactions.

Pilin (PI-1)	Variant	Category*	Major characteristics and target	References
***A:* RrgA_** PBD: 2WW8	RrgA recombinant; bacteria associated RrgA (P1-variants)	I	RrgA binds to human respiratory epithelial A594 cells (independent of P1-RrgB polymerization); P1-Rrg A mediates colonization of the upper respiratory tract in mice.	Nelson et al., 2007
	recombinant RrgA; purified P1 (TIGR4 WT)	I	Dose-dependent binding of RrgA monomer and isolated pili to ECM components (fibronectin, collagen I, laminin)/ELISA.	Hilleringmann et al., 2008
	recombinant RrgA FL and individual RrgA domains	I°	D3/D4 – domain binding mechanism of RrgA with ECM fibronectin under force/AFM-based single molecule force spectroscopy.	Becke et al., 2018
	RrgA recombinant; bacteria associated RrgA (P1-variants)	I/II	RrgA mediated binding to CR3 and CR3 dependent uptake of RrgA containing pneumococci by macrophages; RrgA-expression promotes systemic pneumococcal spread and virulent in mice expressing CR3.	Orrskog et al., 2012
	RrgA recombinant; bacteria associated RrgA (P1-variants)	I	P1 functions as TLR2 agonist – with major contribution of RrgA D3 – in human epithelial cells. Rekombinant RrgA oligomers show increased TLR 2 activation. RrgA D3 is involved in pneumococcal mediated TLR2-activation, TNF-α induction and virulence in a mouse model of infection.	Basset et al., 2013
	RrgA recombinant; bacteria associated RrgA (P1-variants)	I/III	RrgA-containing P1 facilitate passage of *S. pneumoniae* through the blood-brain barrier (BBB) to cause lethal meningitis. Favored variants passing BBB of mice are spherical, single, P1-RrgA + pneumococci.	Iovino et al., 2016a
			Binding of RrgA to BBB endothelial receptors (PECAM-1 and pIgR) promotes bacterial entry and meningitis development.	Iovino et al., 2017
			Interaction of RrgA and pneumolysin with β-actin stimulate meningitis related neuronal death.	Tabusi et al., 2020
	Bacteria associated RrgA (P1-variants)	I + IV	Potential role of RrgA in biofilm formation / promotion of inter-bacterial interaction.	Muñoz-Elías et al., 2008
	RrgA recombinant; bacteria associated RrgA (P1-variants)	I + n/a	Role of RrgA as lectin targeting different host glycosylation pattern.	Day et al., 2017
***B:* Rrg B_** PDB: 3RPK/2Y1V	RrgB recombinant; bacteria P1-variants	I + n/a	Role of RrgB as lectin targeting different host glycosylation pattern.	Day et al., 2017
	Recombinant RrgB FL and RrgB ΔD3 variants	I°	RrgB binds to ECM collagen I in a force-dependent manner and depends on the orientation of lateral RrgB D3-domain and the respective position of the collagen fibrils.	(Becke et al., 2019
***C:* RrgC_** PDB: 4OQ1	RrgC recombinant; bacteria P1-variants	I + n/a	Role of RrgC as lectin targeting different host glycosylation pattern.	Day et al., 2017

Category*: I, adhesion to ECM/surfaces/mammalian cells; II, evasion of innate and adaptive immune responses; III, invasion of non-phagocytic host cells by pathogen-directed endocytosis; IV, cell–cell aggregation during biofilm formation.°Interaction under mechanical force conditions; n/a, not available.P1, pilus-1; WT, wild-type; ECM, extracellular matrix; FL, full length; CR3, complement receptor 3; TLR2, Toll-like receptor 2; TNFα, tumor necrosis factor α; PECAM-1, platelet endothelial cell adhesion molecule 1; plgR, polymeric immunoglobulin receptor; AFM, atomic force microscopy.

Polymerized backbone subunits of Gram-positive pili are primarily considered as stabilized and flexible stalk exposing tip based adhesins for proper host interaction. Recent data suggest a specific role for backbone pilins in host interaction, as shown for pili of *Streptococcus pyogenes* (Tsai et al., 2017; Chen et al., 2020). Interestingly, under mechanical load, P1 RrgB was found to strongly interact with human collagen I (Table 1B) not measurable using static ELISA (Hilleringmann et al., 2008; Becke et al., 2019). The particular lateral domain D3 of RrgB (Figure 1-A3) is essential for the observed exceptionally high binding forces and a force-induced bond strengthening, a described property of bacterial adhesins (Herman-Bausier et al., 2018). This implies a discrete functional role of the P1 backbone in host factor interaction. Specific binding characteristics mediated by numerous linearly arranged RrgB molecules within P1 needs further investigation. Broader experimental approaches better mimicking *in vivo* conditions might reveal novel concepts of host interaction, also mediated by backbone subunits of Gram-positive pili.

A common class of host target structures of many bacterial adhesins are glycoconjugates. Work by Day et al. (2017) describe P1 subunits as new pneumococcal lectins binding several glycosylation pattern: major P1 adhesin RrgA shows the broadest glycan binding repertoire (namely maltose, cellobiose, α/β linked galactose, blood group A and H antigens) when compared to RrgB and RrgC specificities. Interestingly, screening glycosylation targets of P1 subunits also identified non-human and non-mammalian patterns, assigning an even larger spectrum of potential P1 interactions (Day et al., 2017).

Apart from its potential role as lectin (Day et al., 2017), no further host interaction factor for P1 anchor RrgC is known (Table 1C).

The multiplicity of described P1-mediated host interaction scenarios makes P1 a pneumococcal virulence factor with strong impact on the pathogenesis of *S. pneumoniae*. P1 was shown to contribute to initial steps of colonization but also promotes invasion and spreading within the host (Table 1). Despite obvious advantages, P1 was identified in only a relatively small proportion of pneumococcal clinical isolates (−30%) belonging to few clonal complexes (Dzaraly et al., 2020) and PI-1 positive strains show a biphasic P1 expression pattern (Angelis et al., 2011). This implies potential limitations for pneumococcal fitness and virulence related to P1 expression: synthesis of high molecular weight architecture of P1 is complex (Figure 1) and might be tightly regulated by the bacterial metabolism. Iovino et al. (2020) propose two trade-off mechanisms explaining the relatively low % of P1 expressing strains: a high P1 induced host immunogenicity related to surface exposed P1 filaments that may also prevent recolonization and a low host transmissibility due to P1 adhesive properties. Additionally *S. pneumoniae* produces a variety of surface exposed, virulence related factors promoting various host interactions (Mitchell and Mitchell, 2010; Hilleringmann et al., 2015) that may substitute similar P1 mediated functions (e.g., PavA – fibronectin recognition). Importantly epidemiological data show that strain isolates that contain PI-1 are often associated with successful pneumococcal lineages and antimicrobial resistance pattern (Dzaraly et al., 2020), suggesting P1 as a particular tool among the diversity of pneumococcal virulence factors.

## Future Direction and Concluding Remarks

One and a half decades after the first description of *S. pneumoniae* P1 a considerable amount of data characterizes these very long, thin and highly stable surface appendages as evolutionary optimized subunit assemblies that promote pneumococcal virulence mediating multifunctional interactions in different host niches. Although the main components essential for P1 formation and the resulting architecture are well described, details of the spatio-temporal interplay during P1 assembly on the bacterial surface and their regulation (“P1-assembly machinery”) needs further analysis, applying, e.g., novel high resolution microscopic approaches. In addition, a more complete picture of P1 functional aspects and the *in vivo* relevance demands a greater focus on complementary experimental approaches mimicking host environments [e.g., mechanical force- conditions (Dufrêne and Persat, 2020; Viela et al., 2020)] in addition to suitable *in vivo* models. These data will allow a more profound evaluation of P1 mediated bacterial fitness benefits with related costs of P1 expression and the risk of P1 induced adverse host immune responses. Promising antigenic properties of P1 subunits were demonstrated in several animal studies (Gianfaldoni et al., 2007; Moschioni et al., 2010; Gentile et al., 2011; Harfouche et al., 2012). Although P1 is found in only ∼ 30% of clinical pneumococcal isolates, its complex involvement with bacterial virulence, and data implying an association of P1 with antibiotic resistance and evolving non-vaccine serotypes (Dzaraly et al., 2020) make P1 subunits interesting protein-based vaccine candidates. Rational antigen (AG) design and innovative formulation strategies might enable a future non-serotype-dependent, efficient vaccination against *S. pneumoniae*, containing several protective pneumococcal protein-AGs, potentially in combination with known polysaccharide-conjugate AG designs (Converso et al., 2020). A direct interference with P1 function, like small molecule or antibody-mediated inhibition of PI-1 sortase activity, blocking of specific P1 epitopes involved in host interaction (Amerighi et al., 2016) or destabilizing P1 backbone subunits to reduce P1 functionality in host environment as shown for Spy0128, the major pilin from the Gram-positive human pathogen *S. pyogenes* (Rivas-Pardo et al., 2018) constitute further potential strategies to reduce P1-mediated virulence. In addition, understanding P1 on a molecular level also enabled the design of innovative bioconjugation tools (Bonnet et al., 2017) and demonstrates the many facets of this fascinating structure.

## Author Contributions

SN and MH wrote the manuscript and performed critical revision of the work. Both authors approved the submitted version.

## Conflict of Interest

The authors declare that the research was conducted in the absence of any commercial or financial relationships that could be construed as a potential conflict of interest.

## References

[B1] Amerighi F. Valeri M. Donnarumma D. Maccari S. Moschioni M. Taddei A. (2016). Identification of a monoclonal antibody against pneumococcal pilus 1 ancillary protein impairing bacterial adhesion to human epithelial cells. *J. Infect. Dis.* 213 516–522. 10.1093/infdis/jiv461 26401026

[B2] Angelis G. de, Moschioni M. Muzzi A. Pezzicoli A. Censini S. (2011). The *Streptococcus pneumoniae* pilus-1 displays a biphasic expression pattern. *PLoS One* 6:e21269. 10.1371/journal.pone.0021269 21731688 PMC3120856

[B3] Bagnoli F. Moschioni M. Donati C. Dimitrovska V. Ferlenghi I. Facciotti C. (2008). A second pilus type in *Streptococcus pneumoniae* is prevalent in emerging serotypes and mediates adhesion to host cells. *J. Bacteriol.* 190 5480–5492. 10.1128/jb.00384-08 18515415 PMC2493256

[B4] Baker E. N. Squire C. J. Young P. G. (2015). Self-generated covalent cross-links in the cell-surface adhesins of Gram-positive bacteria. *Biochem. Soc. Trans.* 43 787–794. 10.1042/bst20150066 26517883

[B5] Barocchi M. A. Ries J. Zogaj X. Hemsley C. Albiger B. Kanth A. (2006). A pneumococcal pilus influences virulence and host inflammatory responses. *Proc. Natl. Acad. Sci. U.S.A.* 103 2857–2862. 10.1073/pnas.0511017103 16481624 PMC1368962

[B6] Basset A. Herd M. Daly R. Dove S. L. Malley R. (2017). The Pneumococcal type 1 Pilus genes are thermoregulated and are repressed by a member of the snf2 protein family. *J. Bacteriol.* 199:e00078-17. 10.1128/JB.00078-17 PMC5512219 28507246

[B7] Basset A. Turner K. H. Boush E. Sayeed S. Dove S. L. Malley R. (2011). Expression of the type 1 pneumococcal pilus is bistable and negatively regulated by the structural component RrgA. *Infect. Immun.* 79 2974–2983. 10.1128/iai.05117-11 21576325 PMC3147576

[B8] Basset A. Turner K. H. Boush E. Sayeed S. Dove S. L. Malley R. (2012). An epigenetic switch mediates bistable expression of the type 1 pilus genes in *Streptococcus pneumoniae*. *J. Bacteriol.* 194 1088–1091. 10.1128/jb.06078-11 22194460 PMC3294793

[B9] Basset A. Zhang F. Benes C. Sayeed S. Herd M. Thompson C. (2013). Toll-like receptor (TLR) 2 mediates inflammatory responses to oligomerized RrgA pneumococcal pilus type 1 protein. *J. Biol. Chem.* 288 2665–2675. 10.1074/jbc.m112.398875 23233677 PMC3554933

[B10] Becke T. D. Ness S. Gürster R. Schilling A. F. Di Guilmi A.-M. Sudhop S. (2018). Single molecule force spectroscopy reveals two-domain binding mode of pilus-1 tip protein RrgA of *Streptococcus pneumoniae* to Fibronectin. *ACS Nano* 12 549–558. 10.1021/acsnano.7b07247 29298375

[B11] Becke T. D. Ness S. Kaufmann B. K. Hartmann B. Schilling A. F. Sudhop S. (2019). Pilus-1 backbone protein RrgB of *Streptococcus pneumoniae* binds collagen I in a force-dependent way. *ACS Nano* 13 7155–7165. 10.1021/acsnano.9b02587 31184856

[B12] Binsker U. Lees J. A. Hammond A. J. Weiser J. N. (2020). Immune exclusion by naturally acquired secretory IgA against pneumococcal pilus-1. *J. Clin. Invest.* 130 927–941. 10.1172/jci132005 31687974 PMC6994158

[B13] Bonnet J. Cartannaz J. Tourcier G. Contreras-Martel C. Kleman J. P. Morlot C. (2017). Autocatalytic association of proteins by covalent bond formation: a Bio Molecular Welding toolbox derived from a bacterial adhesin. *Sci. Rep.* 7:43564. 10.1038/srep43564 PMC5333627 28252635

[B14] Chang C. Wu C. Osipiuk J. Siegel S. D. Zhu S. Liu X. (2019). Cell-to-cell interaction requires optimal positioning of a pilus tip adhesin modulated by gram-positive transpeptidase enzymes. *Proc. Natl. Acad. Sci. U.S.A.* 116 18041–18049. 10.1073/pnas.1907733116 31427528 PMC6731673

[B15] Chen Y.-H. Li S.-H. Yang Y.-C. Hsu S.-H. Nizet V. Chang Y.-C. (2020). T4 Pili promote colonization and immune evasion phenotypes of nonencapsulated M4 *Streptococcus pyogenes*. *mBio* 11:e01580-20. 10.1128/mBio.01580-20 PMC7374061 32694142

[B16] Converso T. R. Assoni L. André G. O. Darrieux M. Leite L. C. C. (2020). The long search for a serotype independent pneumococcal vaccine. *Expert Rev. Vaccines* 19 57–70. 10.1080/14760584.2020.1711055 31903805

[B17] Day C. J. Paton A. W. Harvey R. M. Hartley-Tassell L. E. Seib K. L. Tiralongo J. (2017). Lectin activity of the pneumococcal pilin proteins. *Sci. Rep.* 7:17784. 10.1038/s41598-017-17850-9 PMC5736695 29259314

[B18] Dufrêne Y. F. Persat A. (2020). Mechanomicrobiology: how bacteria sense and respond to forces. *Nat. Rev. Microbiol.* 18 227–240. 10.1038/s41579-019-0314-2 31959911

[B19] Dzaraly N. D. Muthanna A. Mohd Desa M. N. Taib N. M. Masri S. N. Rahman N. I. A. (2020). Pilus islets and the clonal spread of piliated *Streptococcus pneumoniae*: a review. *Int. J. Med. Microbiol.* 310:151449. 10.1016/j.ijmm.2020.151449 33092697

[B20] Echelman D. J. Alegre-Cebollada J. Badilla C. L. Chang C. Ton-That H. Fernández J. M. (2016). CnaA domains in bacterial pili are efficient dissipaters of large mechanical shocks. *Proc. Natl. Acad. Sci. U.S.A.* 113 2490–2495. 10.1073/pnas.1522946113 26884173 PMC4780631

[B21] El Mortaji L. Contreras-Martel C. Moschioni M. Ferlenghi I. Manzano C. Vernet T. (2012a). The full-length *Streptococcus pneumoniae* major pilin RrgB crystallizes in a fibre-like structure, which presents the D1 isopeptide bond and provides details on the mechanism of pilus polymerization. *Biochem. J.* 441 833–841. 10.1042/bj20111397 22013894

[B22] El Mortaji L. Fenel D. Vernet T. Di Guilmi A. M. (2012b). Association of RrgA and RrgC into the *Streptococcus pneumoniae* pilus by sortases C-2 and C-3. *Biochemistry* 51 342–352. 10.1021/bi201591n 22122269

[B23] El Mortaji L. Terrasse R. Dessen A. Vernet T. Di Guilmi A. M. (2010). Stability and assembly of pilus subunits of *Streptococcus pneumoniae*. *J. Biol. Chem.* 285 12405–12415. 10.1074/jbc.m109.082776 20147289 PMC2852978

[B24] Fälker S. Nelson A. L. Morfeldt E. Jonas K. Hultenby K. Ries J. (2008). Sortase-mediated assembly and surface topology of adhesive pneumococcal pili. *Mol. Microbiol.* 70 595–607. 10.1111/j.1365-2958.2008.06396.x 18761697 PMC2680257

[B25] Figueira M. Moschioni M. Angelis G. de, Barocchi M. Sabharwal V. (2014). Variation of pneumococcal Pilus-1 expression results in vaccine escape during Experimental Otitis Media EOM. *PLoS One* 9:e83798. 10.1371/journal.pone.0083798 24421906 PMC3885439

[B26] Gentile M. A. Melchiorre S. Emolo C. Moschioni M. Gianfaldoni C. Pancotto L. (2011). Structural and functional characterization of the *Streptococcus pneumoniae* RrgB pilus backbone D1 domain. *J. Biol. Chem.* 286 14588–14597. 10.1074/jbc.m110.202739 21367860 PMC3077656

[B27] Gianfaldoni C. Censini S. Hilleringmann M. Moschioni M. Facciotti C. Pansegrau W. (2007). *Streptococcus pneumoniae* pilus subunits protect mice against lethal challenge. *Infect. Immun.* 75 1059–1062. 10.1128/iai.01400-06 17145945 PMC1828493

[B28] Gómez-Mejia A. Gámez G. Hirschmann S. Kluger V. Rath H. Böhm S. (2018). Pneumococcal metabolic adaptation and colonization are regulated by the two-component regulatory system 08. *mSphere* 3:e00165-18. 10.1128/mSphere.00165-18 PMC5956151 29769380

[B29] Harfouche C. Filippini S. Gianfaldoni C. Ruggiero P. Moschioni M. Maccari S. (2012). RrgB321, a fusion protein of the three variants of the pneumococcal pilus backbone RrgB, is protective in vivo and elicits opsonic antibodies. *Infect. Immun.* 80 451–460. 10.1128/iai.05780-11 22083702 PMC3255673

[B30] Hava D. L. LeMieux J. Camilli A. (2003). From nose to lung: the regulation behind *Streptococcus pneumoniae* virulence factors. *Mol. Microbiol.* 50 1103–1110. 10.1046/j.1365-2958.2003.03764.x 14622402 PMC2791164

[B31] Hendrickx A. P. A. Budzik J. M. Oh S.-Y. Schneewind O. (2011). Architects at the bacterial surface - sortases and the assembly of pili with isopeptide bonds. *Nat. Rev. Microbiol.* 9 166–176. 10.1038/nrmicro2520 21326273

[B32] Herbert J. A. Mitchell A. M. Mitchell T. J. (2015). A serine-threonine kinase (StkP) regulates expression of the Pneumococcal pilus and modulates bacterial adherence to human epithelial and endothelial cells *in vitro*. *PLoS One* 10:e0127212. 10.1371/journal.pone.0127212 26090876 PMC4474723

[B33] Herman-Bausier P. Labate C. Towell A. M. Derclaye S. Geoghegan J. A. Dufrêne Y. F. (2018). Staphylococcus aureus clumping factor A is a force-sensitive molecular switch that activates bacterial adhesion. *Proc. Natl. Acad. Sci. U.S.A.* 115 5564–5569. 10.1073/pnas.1718104115 29735708 PMC6003445

[B34] Hilleringmann M. Giusti F. Baudner B. C. Masignani V. Covacci A. Rappuoli R. (2008). Pneumococcal pili are composed of protofilaments exposing adhesive clusters of Rrg A. *PLoS Pathog.* 4:e1000026. 10.1371/journal.ppat.1000026 18369475 PMC2265430

[B35] Hilleringmann M. Kohler S. Gámez G. Hammerschmidt S. (2015). “Pneumococcal pili and adhesins,” in *Streptococcus pneumoniae Molecular Mechanisms of Host-Pathogen Interactions.* eds Brown J. Hammerschmidt S. Orihuela C. (London: Elsevier).

[B36] Hilleringmann M. Ringler P. Müller S. A. Angelis G. de, Rappuoli R. (2009). Molecular architecture of *Streptococcus pneumoniae* TIGR4 pili. *EMBO J.* 28 3921–3930. 10.1038/emboj.2009.360 19942854 PMC2797065

[B37] Hospenthal M. K. Costa T. R. D. Waksman G. (2017). A comprehensive guide to pilus biogenesis in Gram-negative bacteria. *Nat. Rev. Microbiol.* 15 365–379. 10.1038/nrmicro.2017.40 28496159

[B38] Iovino F. Engelen-Lee J.-Y. Brouwer M. van de Beek D. van der Ende A. Valls Seron M. (2017). pIgR and PECAM-1 bind to pneumococcal adhesins RrgA and PspC mediating bacterial brain invasion. *J. Exp. Med.* 214 1619–1630. 10.1084/jem.20161668 28515075 PMC5461002

[B39] Iovino F. Hammarlöf D. L. Garriss G. Brovall S. Nannapaneni P. Henriques-Normark B. (2016a). Pneumococcal meningitis is promoted by single cocci expressing pilus adhesin RrgA. *J. Clin. Invest.* 126 2821–2826. 10.1172/jci84705 27348589 PMC4966305

[B40] Iovino F. Nannapaneni P. Henriques-Normark B. Normark S. (2020). The impact of the ancillary pilus-1 protein RrgA of *Streptococcus pneumoniae* on colonization and disease. *Mol. Microbiol.* 113 650–658. 10.1111/mmi.14451 32185835

[B41] Iovino F. Seinen J. Henriques-Normark B. van Dijl J. M. (2016b). How Does *Streptococcus pneumoniae* invade the brain? *Trends Microbiol.* 24 307–315. 10.1016/j.tim.2015.12.012 26804733

[B42] Izoré T. Contreras-Martel C. El Mortaji L. Manzano C. Terrasse R. Vernet T. (2010). [Duplikat] Structural basis of host cell recognition by the pilus adhesin from *Streptococcus pneumoniae*. *Structure* 18 106–115. 10.1016/j.str.2009.10.019 20152157

[B43] Jacobitz A. W. Naziga E. B. Yi S. W. McConnell S. A. Peterson R. Jung M. E. (2016). The “Lid” in the *Streptococcus pneumoniae* SrtC1 sortase adopts a rigid structure that regulates substrate access to the active site. *J. Phys. Chem. B* 120 8302–8312. 10.1021/acs.jpcb.6b01930 27109553 PMC5097456

[B44] Kang H. J. Coulibaly F. Clow F. Proft T. Baker E. N. (2007). Stabilizing isopeptide bonds revealed in gram-positive bacterial pilus structure. *Science* 318 1625–1628. 10.1126/science.1145806 18063798

[B45] Khare B. Narayana S. V. L. (2017). Pilus biogenesis of Gram-positive bacteria: roles of sortases and implications for assembly. *Protein Sci.* 26 1458–1473. 10.1002/pro.3191 28493331 PMC5521585

[B46] Kreikemeyer B. Gámez G. Margarit I. Giard J.-C. Hammerschmidt S. Hartke A. (2011). Genomic organization, structure, regulation and pathogenic role of pilus constituents in major pathogenic *Streptococci* and *Enterococci*. *Int. J. Med. Microbiol.* 301 240–251. 10.1016/j.ijmm.2010.09.003 21112248

[B47] Krishnan V. Gaspar A. H. Ye N. Mandlik A. Ton-That H. Narayana S. V. L. (2007). An IgG-like domain in the minor pilin GBS52 of Streptococcus agalactiae mediates lung epithelial cell adhesion. *Structure* 15 893–903. 10.1016/j.str.2007.06.015 17697995 PMC2844079

[B48] Laurenceau R. Péhau-Arnaudet G. Baconnais S. Gault J. Malosse C. Dujeancourt A. (2013). A type IV pilus mediates DNA binding during natural transformation in *Streptococcus pneumoniae*. *PLoS Pathog.* 9:e1003473. 10.1371/journal.ppat.1003473 23825953 PMC3694846

[B49] LeMieux J. Hava D. L. Basset A. Camilli A. (2006). RrgA and RrgB are components of a multisubunit pilus encoded by the *Streptococcus pneumoniae* rlrA pathogenicity islet. *Infect. Immun.* 74 2453–2456. 10.1128/iai.74.4.2453-2456.2006 16552078 PMC1418942

[B50] LeMieux J. Woody S. Camilli A. (2008). Roles of the sortases of *Streptococcus pneumoniae* in assembly of the RlrA pilus. *J. Bacteriol.* 190 6002–6013. 10.1128/jb.00379-08 18606733 PMC2519520

[B51] Loughran A. J. Orihuela C. J. Tuomanen E. I. (2019). *Streptococcus pneumoniae*: invasion and inflammation. *Microbiol. Spectr.* 7:10.1128/microbiolspec.GPP3-0004-2018. 10.1128/microbiolspec.gpp3-0004-2018 PMC6422050 30873934

[B52] Mandlik A. Das A. Ton-That H. (2008). The molecular switch that activates the cell wall anchoring step of pilus assembly in gram-positive bacteria. *Proc. Natl. Acad. Sci. U.S.A.* 105 14147–14152. 10.1073/pnas.0806350105 18779588 PMC2734112

[B53] Manzano C. Contreras-Martel C. El Mortaji L. Izoré T. Fenel D. Vernet T. (2008). Sortase-mediated pilus fiber biogenesis in *Streptococcus pneumoniae*. *Structure* 16 1838–1848. 10.1016/j.str.2008.10.007 19081060

[B54] Manzano C. Izoré T. Job V. Di Guilmi A. M. Dessen A. (2009). Sortase activity is controlled by a flexible lid in the pilus biogenesis mechanism of gram-positive pathogens. *Biochemistry* 48 10549–10557. 10.1021/bi901261y 19810750

[B55] Mitchell A. M. Mitchell T. J. (2010). *Streptococcus pneumoniae*: virulence factors and variation. *Clin. Microbiol. Infect.* 16 411–418. 10.1111/j.1469-0691.2010.03183.x 20132250

[B56] Moschioni M. Emolo C. Biagini M. Maccari S. Pansegrau W. Donati C. (2010). The two variants of the *Streptococcus pneumoniae* pilus 1 RrgA adhesin retain the same function and elicit cross-protection in vivo. *Infect. Immun.* 78 5033–5042. 10.1128/iai.00601-10 20823200 PMC2981310

[B57] Muhammad M. H. Idris A. L. Fan X. Guo Y. Yu Y. Jin X. (2020). Beyond risk: bacterial biofilms and their regulating approaches. *Front. Microbiol.* 11:928. 10.3389/fmicb.2020.00928 32508772 PMC7253578

[B58] Muñoz-Elías E. J. Marcano J. Camilli A. (2008). Isolation of *Streptococcus pneumoniae* biofilm mutants and their characterization during nasopharyngeal colonization. *Infect. Immun.* 76 5049–5061. 10.1128/iai.00425-08 18794289 PMC2573321

[B59] Muschiol S. Aschtgen M.-S. Nannapaneni P. Henriques-Normark B. (2019). Gram-positive type IV pili and competence. *Microbiol. Spectr.* 7 129–135. 10.1128/microbiolspec.psib-0011-2018 PMC11588153 30737914

[B60] Naziga E. B. Wereszczynski J. (2017). Molecular mechanisms of the binding and specificity of *Streptococcus pneumoniae* Sortase C enzymes for pilin subunits. *Sci. Rep.* 7:13119. 10.1038/s41598-017-13135-3 PMC5640630 29030637

[B61] Neiers F. Madhurantakam C. Fälker S. Manzano C. Dessen A. Normark S. (2009a). Two crystal structures of pneumococcal pilus sortase C provide novel insights into catalysis and substrate specificity. *J. Mol. Biol.* 393 704–716. 10.1016/j.jmb.2009.08.058 19729023

[B62] Neiers F. Madhurantakam C. Fälker S. Normark S. Henriques-Normark B. Achour A. (2009b). Cloning, expression, purification, crystallization and preliminary X-ray analysis of the pilus-associated sortase C from *Streptococcus pneumoniae*. *Acta Crystallogr. Sect. F Struct. Biol. Cryst. Commun.* 65 55–58. 10.1107/s1744309108040025 19153457 PMC2628848

[B63] Nelson A. L. Ries J. Bagnoli F. Dahlberg S. Fälker S. Rounioja S. (2007). RrgA is a pilus-associated adhesin in *Streptococcus pneumoniae*. *Mol. Microbiol.* 66 329–340. 10.1111/j.1365-2958.2007.05908.x 17850254 PMC2170534

[B64] Orrskog S. Rounioja S. Spadafina T. Gallotta M. Norman M. Hentrich K. (2012). Pilus adhesin RrgA interacts with complement receptor 3, thereby affecting macrophage function and systemic pneumococcal disease. *mBio* 4:e00535-12. 10.1128/mBio.00535-12 PMC3531807 23269830

[B65] Pancotto L. Angelis G. de, Bizzarri E. Barocchi M. A. Del Giudice G. (2013). Expression of the *Streptococcus pneumoniae* pilus-1 undergoes on and off switching during colonization in mice. *Sci. Rep.* 3:2040. 10.1038/srep02040 PMC3687230 23784148

[B66] Paterson N. G. Baker E. N. (2011). Structure of the full-length major pilin from *Streptococcus pneumoniae*: implications for isopeptide bond formation in gram-positive bacterial pili. *PLoS One* 6:e22095. 10.1371/journal.pone.0022095 21760959 PMC3132780

[B67] Ramirez N. A. Das A. Ton-That H. (2020). New paradigms of pilus assembly mechanisms in gram-positive Actinobacteria. *Trends Microbiol.* 28 999–1009. 10.1016/j.tim.2020.05.008 32499101 PMC7657965

[B68] Rivas-Pardo J. A. Badilla C. L. Tapia-Rojo R. Alonso-Caballero Á. Fernández J. M. (2018). Molecular strategy for blocking isopeptide bond formation in nascent pilin proteins. *Proc. Natl. Acad. Sci. U.S.A.* 115 9222–9227. 10.1073/pnas.1807689115 30150415 PMC6140542

[B69] Shaik M. M. Maccagni A. Tourcier G. Di Guilmi A. M. Dessen A. (2014). Structural basis of pilus anchoring by the ancillary pilin RrgC of *Streptococcus pneumoniae*. *J. Biol. Chem.* 289 16988–16997. 10.1074/jbc.m114.555854 24755220 PMC4059141

[B70] Spirig T. Weiner E. M. Clubb R. T. (2011). Sortase enzymes in Gram-positive bacteria. *Mol. Microbiol.* 82 1044–1059. 10.1111/j.1365-2958.2011.07887.x 22026821 PMC3590066

[B71] Spraggon G. Koesema E. Scarselli M. Malito E. Biagini M. Norais N. (2010). Supramolecular organization of the repetitive backbone unit of the *Streptococcus pneumoniae* pilus. *PLoS One* 5:e10919. 10.1371/journal.pone.0010919 20559564 PMC2886109

[B72] Subramanian K. Henriques-Normark B. Normark S. (2019). Emerging concepts in the pathogenesis of the *Streptococcus pneumoniae*: from nasopharyngeal colonizer to intracellular pathogen. *Cell. Microbiol.* 21:e13077. 10.1111/cmi.13077 PMC6899785 31251447

[B73] Tabusi M. Thorsdottir S. Lysandrou M. Narciso A. R. Minoia M. Henriques-Normark B. (2020). Neuronal death in pneumococcal meningitis is triggered by pneumolysin and pilus-1 interactions with β-actin. *bioRxiv* [Preprint]. 10.1101/2020.08.20.258681 PMC7990213 33760879

[B74] Telford J. L. Barocchi M. A. Margarit I. Rappuoli R. Grandi G. (2006). Pili in gram-positive pathogens. *Nat. Rev. Microbiol.* 4 509–519. 16778837 10.1038/nrmicro1443

[B75] Tsai J.-Y. C. Loh J. M. S. Clow F. Lorenz N. Proft T. (2017). The Group A Streptococcus serotype M2 pilus plays a role in host cell adhesion and immune evasion. *Mol. Microbiol.* 103 282–298. 10.1111/mmi.13556 27741558

[B76] Viela F. Mathelié-Guinlet M. Viljoen A. Dufrêne Y. F. (2020). What makes bacterial pathogens so sticky? *Mol. Microbiol.* 113 683–690. 10.1111/mmi.14448 31916325

[B77] Young P. G. Moreland N. J. Loh J. M. Bell A. Atatoa Carr P. Proft T. (2014). Structural conservation, variability, and immunogenicity of the T6 backbone pilin of serotype M6 *Streptococcus pyogenes*. *Infect. Immun.* 82 2949–2957. 24778112 10.1128/IAI.01706-14 PMC4097639

